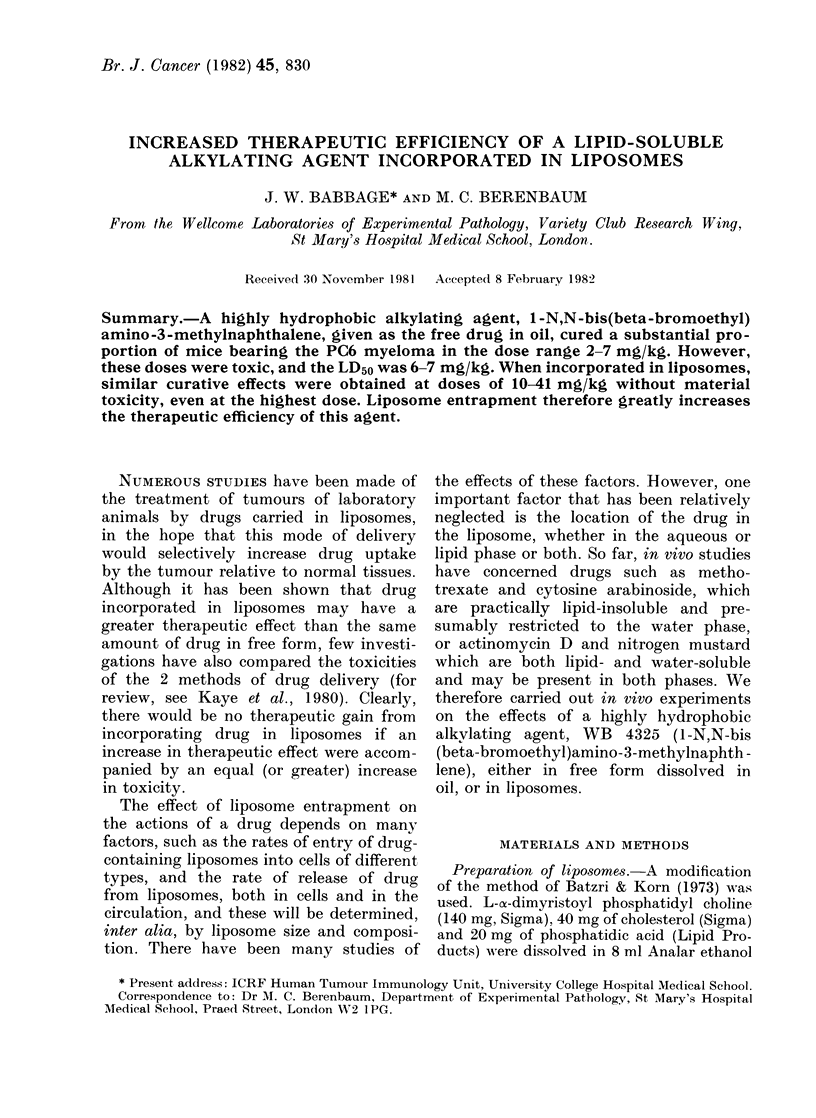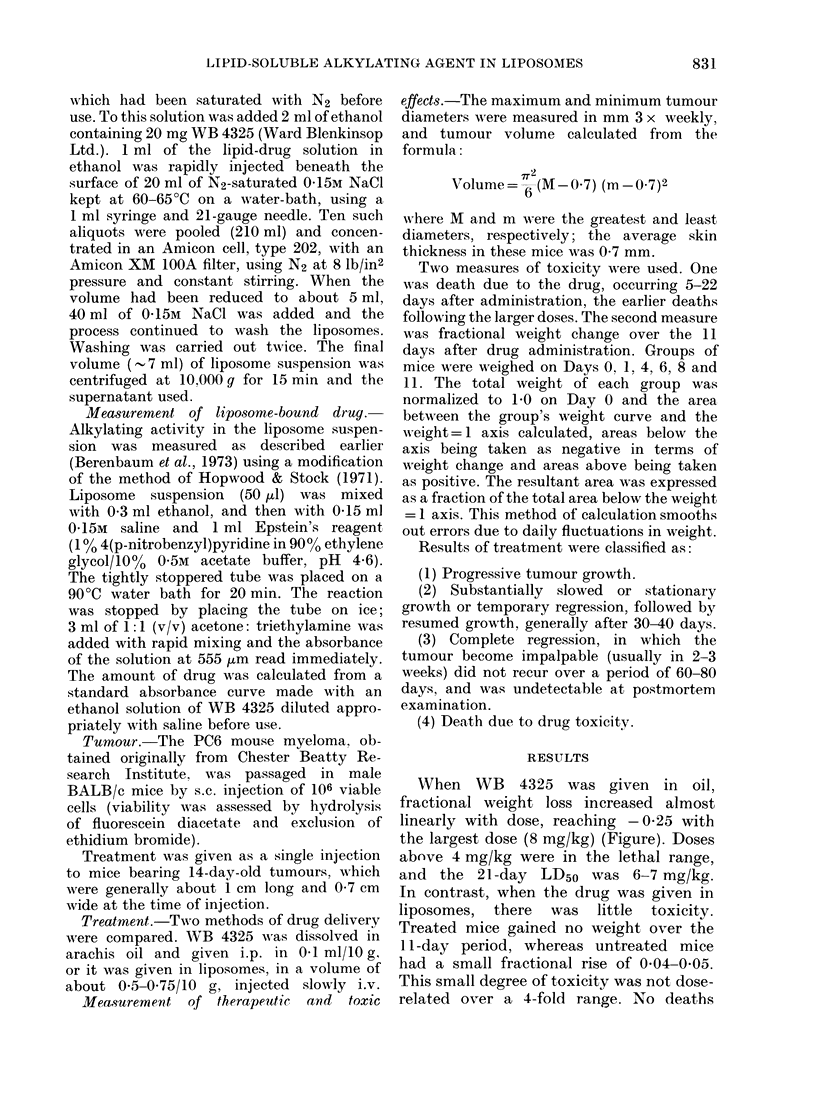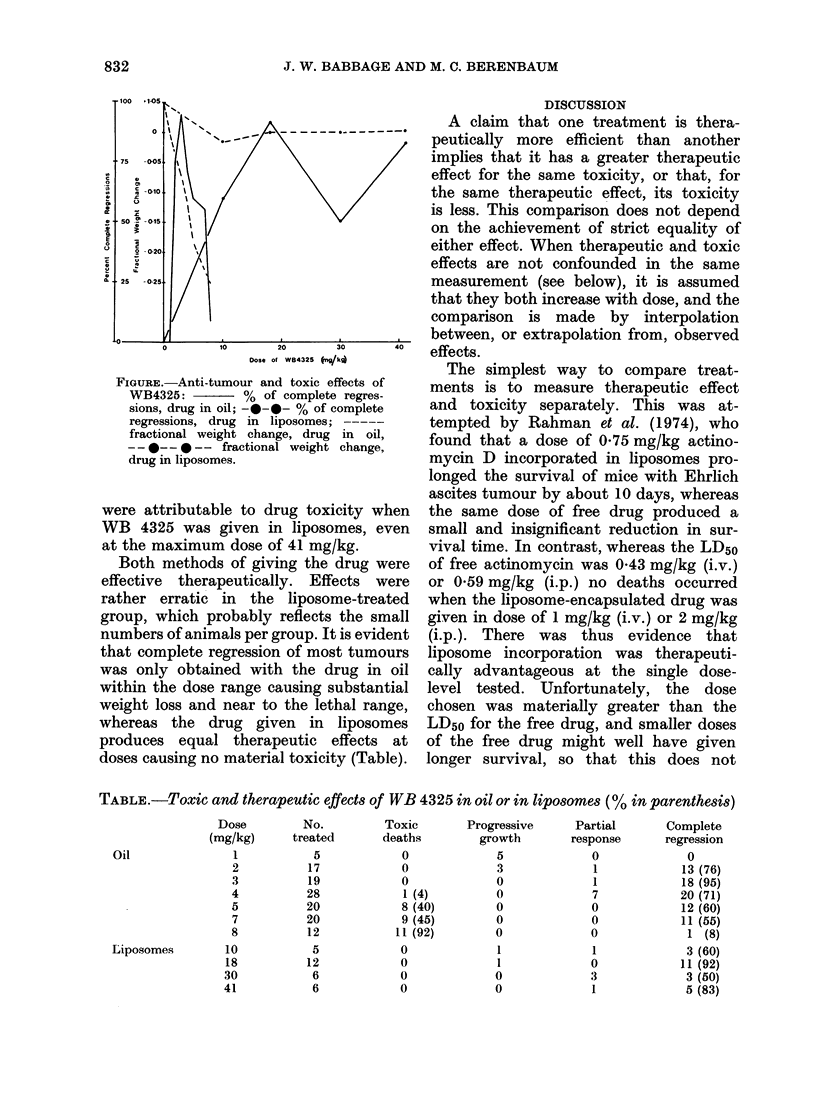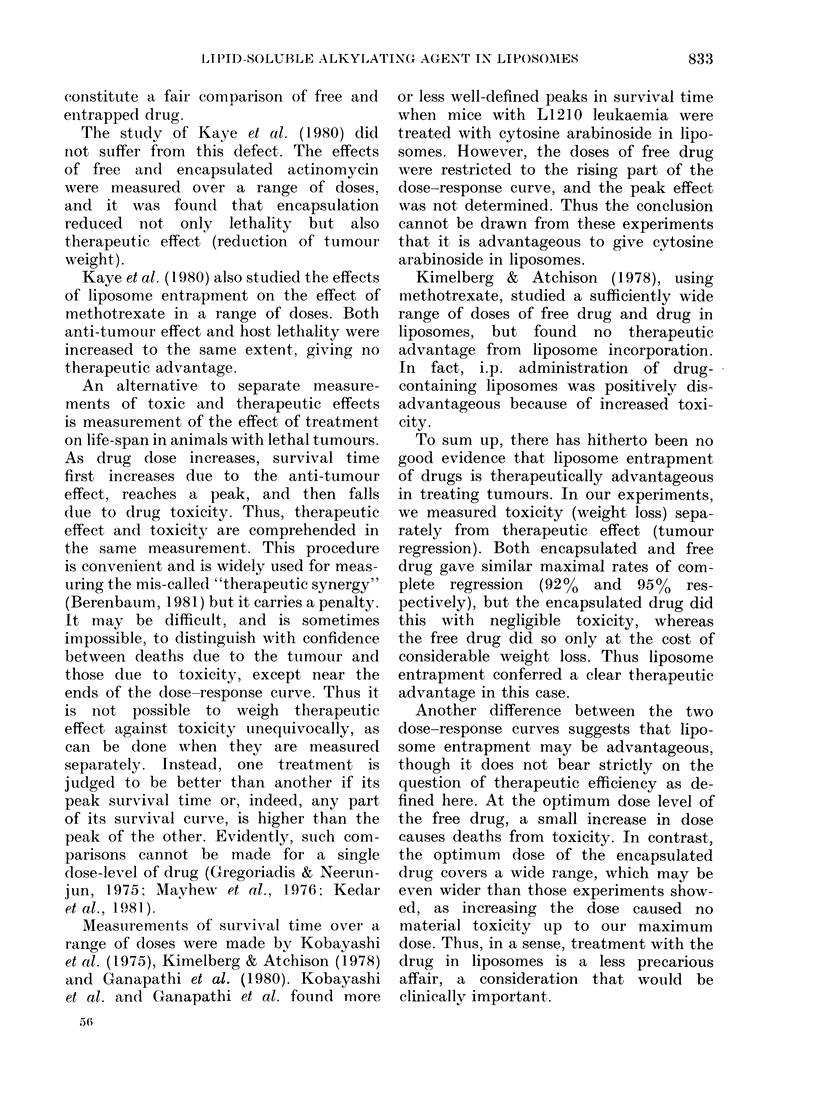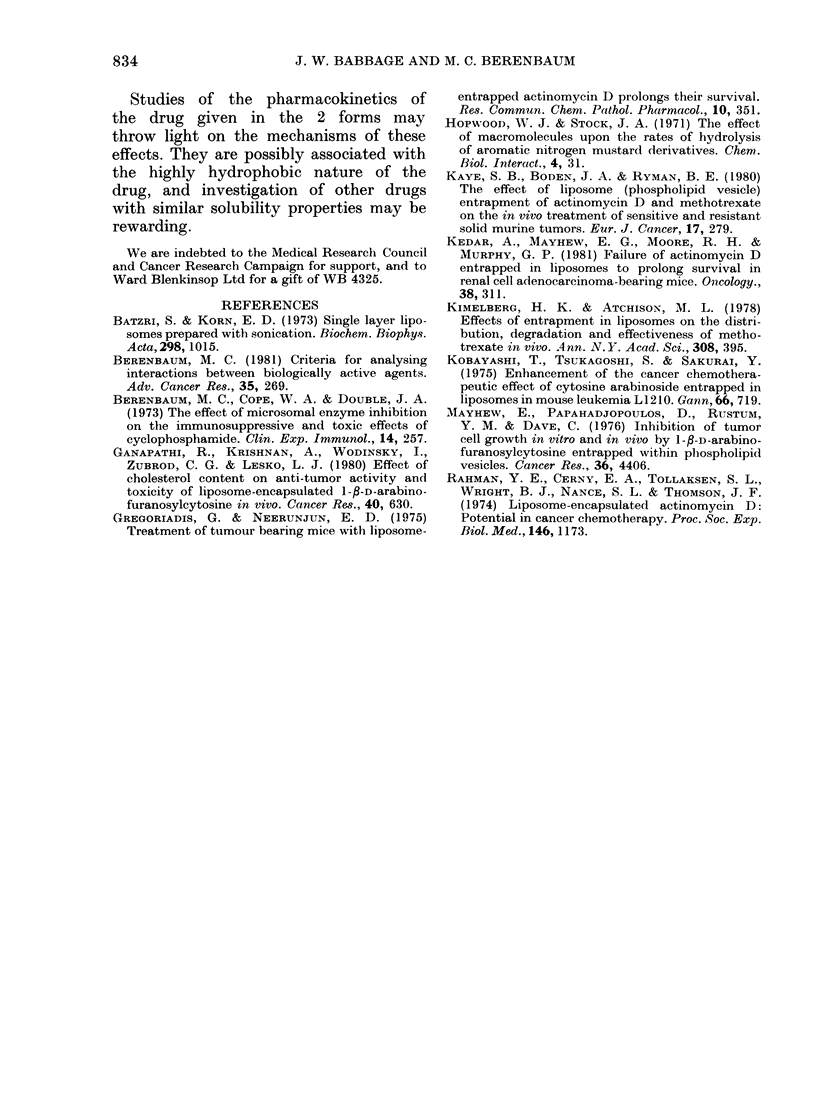# Increased therapeutic efficiency of a lipid-soluble alkylating agent incorporated in liposomes.

**DOI:** 10.1038/bjc.1982.134

**Published:** 1982-06

**Authors:** J. W. Babbage, M. C. Berenbaum

## Abstract

A highly hydrophobic alkylating agent, 1-N,N-bis(beta-bromoethyl) amino-3-methylnaphthalene, given as the free drug in oil, cured a substantial proportion of mice bearing the PC6 myeloma in the dose range 2-7 mg/kg. However, these doses were toxic, and the LD50 was 6-7 mg/kg. When incorporated in liposomes, similar curative effects were obtained at doses of 10-41 mg/kg without material toxicity, even at the highest dose. Liposome entrapment therefore greatly increases the therapeutic efficiency of this agent.


Br. J. Cancer (1982) **45**, 830

### INCREASED THERAPEUTIC EFFICIENCY OF A LIPID-SOLUBLE ALKYLATING AGENT INCORPORATED IN LIPOSOMES

J. W. BABBAGE* AND M. C. BERENBAUM

*From the Wellcome Laboratories of Experimental Pathology, Variety Club Research Wing, St Mary's Hospital Medical School, London.*

Received 30 November 1981 Accepted 8 February 1982

**Summary.—A highly hydrophobic alkylating agent, 1-N,N-bis(beta-bromoethyl) amino-3-methylnaphthalene, given as the free drug in oil, cured a substantial proportion of mice bearing the PC6 myeloma in the dose range 2–7 mg/kg. However, these doses were toxic, and the $LD_{50}$ was 6–7 mg/kg. When incorporated in liposomes, similar curative effects were obtained at doses of 10–41 mg/kg without material toxicity, even at the highest dose. Liposome entrapment therefore greatly increases the therapeutic efficiency of this agent.**

NUMEROUS STUDIES have been made of the treatment of tumours of laboratory animals by drugs carried in liposomes, in the hope that this mode of delivery would selectively increase drug uptake by the tumour relative to normal tissues. Although it has been shown that drug incorporated in liposomes may have a greater therapeutic effect than the same amount of drug in free form, few investigations have also compared the toxicities of the 2 methods of drug delivery (for review, see Kaye *et al.*, 1980). Clearly, there would be no therapeutic gain from incorporating drug in liposomes if an increase in therapeutic effect were accompanied by an equal (or greater) increase in toxicity.

The effect of liposome entrapment on the actions of a drug depends on many factors, such as the rates of entry of drug-containing liposomes into cells of different types, and the rate of release of drug from liposomes, both in cells and in the circulation, and these will be determined, *inter alia*, by liposome size and composition. There have been many studies of the effects of these factors. However, one important factor that has been relatively neglected is the location of the drug in the liposome, whether in the aqueous or lipid phase or both. So far, *in vivo* studies have concerned drugs such as methotrexate and cytosine arabinoside, which are practically lipid-insoluble and presumably restricted to the water phase, or actinomycin D and nitrogen mustard which are both lipid- and water-soluble and may be present in both phases. We therefore carried out *in vivo* experiments on the effects of a highly hydrophobic alkylating agent, WB 4325 (1-N,N-bis (beta-bromoethyl)amino-3-methylnaphth-lene), either in free form dissolved in oil, or in liposomes.

### MATERIALS AND METHODS

*Preparation of liposomes.*—A modification of the method of Batzri & Korn (1973) was used. L-α-dimyristoyl phosphatidyl choline (140 mg, Sigma), 40 mg of cholesterol (Sigma) and 20 mg of phosphatidic acid (Lipid Products) were dissolved in 8 ml Analar ethanol

* Present address: ICRF Human Tumour Immunology Unit, University College Hospital Medical School.
Correspondence to: Dr M. C. Berenbaum, Department of Experimental Pathology, St Mary's Hospital Medical School, Praed Street, London W2 1PG.

which had been saturated with $N_2$ before use. To this solution was added 2 ml of ethanol containing 20 mg WB 4325 (Ward Blenkinsop Ltd.). 1 ml of the lipid-drug solution in ethanol was rapidly injected beneath the surface of 20 ml of $N_2$-saturated 0·15M NaCl kept at 60–65°C on a water-bath, using a 1 ml syringe and 21-gauge needle. Ten such aliquots were pooled (210 ml) and concentrated in an Amicon cell, type 202, with an Amicon XM 100A filter, using $N_2$ at 8 lb/in$^2$ pressure and constant stirring. When the volume had been reduced to about 5 ml, 40 ml of 0·15M NaCl was added and the process continued to wash the liposomes. Washing was carried out twice. The final volume (~7 ml) of liposome suspension was centrifuged at 10,000 *g* for 15 min and the supernatant used.

*Measurement of liposome-bound drug.*—Alkylating activity in the liposome suspension was measured as described earlier (Berenbaum *et al.*, 1973) using a modification of the method of Hopwood & Stock (1971). Liposome suspension (50 μl) was mixed with 0·3 ml ethanol, and then with 0·15 ml 0·15M saline and 1 ml Epstein's reagent (1% 4(p-nitrobenzyl)pyridine in 90% ethylene glycol/10% 0·5M acetate buffer, pH 4·6). The tightly stoppered tube was placed on a 90°C water bath for 20 min. The reaction was stopped by placing the tube on ice; 3 ml of 1:1 (v/v) acetone: triethylamine was added with rapid mixing and the absorbance of the solution at 555 μm read immediately. The amount of drug was calculated from a standard absorbance curve made with an ethanol solution of WB 4325 diluted appropriately with saline before use.

*Tumour.*—The PC6 mouse myeloma, obtained originally from Chester Beatty Research Institute, was passaged in male BALB/c mice by s.c. injection of $10^6$ viable cells (viability was assessed by hydrolysis of fluorescein diacetate and exclusion of ethidium bromide).

Treatment was given as a single injection to mice bearing 14-day-old tumours, which were generally about 1 cm long and 0·7 cm wide at the time of injection.

*Treatment.*—Two methods of drug delivery were compared. WB 4325 was dissolved in arachis oil and given i.p. in 0·1 ml/10 g, or it was given in liposomes, in a volume of about 0·5–0·75/10 g, injected slowly i.v.

*Measurement of therapeutic and toxic effects.*—The maximum and minimum tumour diameters were measured in mm 3× weekly, and tumour volume calculated from the formula:

$$\text{Volume} = \frac{\pi^2}{6}(M - 0{\cdot}7)\,(m - 0{\cdot}7)^2$$

where M and m were the greatest and least diameters, respectively; the average skin thickness in these mice was 0·7 mm.

Two measures of toxicity were used. One was death due to the drug, occurring 5–22 days after administration, the earlier deaths following the larger doses. The second measure was fractional weight change over the 11 days after drug administration. Groups of mice were weighed on Days 0, 1, 4, 6, 8 and 11. The total weight of each group was normalized to 1·0 on Day 0 and the area between the group's weight curve and the weight = 1 axis calculated, areas below the axis being taken as negative in terms of weight change and areas above being taken as positive. The resultant area was expressed as a fraction of the total area below the weight = 1 axis. This method of calculation smooths out errors due to daily fluctuations in weight.

Results of treatment were classified as:

(1) Progressive tumour growth.

(2) Substantially slowed or stationary growth or temporary regression, followed by resumed growth, generally after 30–40 days.

(3) Complete regression, in which the tumour become impalpable (usually in 2–3 weeks) did not recur over a period of 60–80 days, and was undetectable at postmortem examination.

(4) Death due to drug toxicity.

### RESULTS

When WB 4325 was given in oil, fractional weight loss increased almost linearly with dose, reaching −0·25 with the largest dose (8 mg/kg) (Figure). Doses above 4 mg/kg were in the lethal range, and the 21-day $LD_{50}$ was 6–7 mg/kg. In contrast, when the drug was given in liposomes, there was little toxicity. Treated mice gained no weight over the 11-day period, whereas untreated mice had a small fractional rise of 0·04–0·05. This small degree of toxicity was not dose-related over a 4-fold range. No deaths

FIGURE.—Anti-tumour and toxic effects of WB4325: ——— % of complete regressions, drug in oil; -●-●- % of complete regressions, drug in liposomes; ----- fractional weight change, drug in oil, --●--●-- fractional weight change, drug in liposomes.

were attributable to drug toxicity when WB 4325 was given in liposomes, even at the maximum dose of 41 mg/kg.

Both methods of giving the drug were effective therapeutically. Effects were rather erratic in the liposome-treated group, which probably reflects the small numbers of animals per group. It is evident that complete regression of most tumours was only obtained with the drug in oil within the dose range causing substantial weight loss and near to the lethal range, whereas the drug given in liposomes produces equal therapeutic effects at doses causing no material toxicity (Table).

DISCUSSION

A claim that one treatment is therapeutically more efficient than another implies that it has a greater therapeutic effect for the same toxicity, or that, for the same therapeutic effect, its toxicity is less. This comparison does not depend on the achievement of strict equality of either effect. When therapeutic and toxic effects are not confounded in the same measurement (see below), it is assumed that they both increase with dose, and the comparison is made by interpolation between, or extrapolation from, observed effects.

The simplest way to compare treatments is to measure therapeutic effect and toxicity separately. This was attempted by Rahman *et al.* (1974), who found that a dose of 0·75 mg/kg actinomycin D incorporated in liposomes prolonged the survival of mice with Ehrlich ascites tumour by about 10 days, whereas the same dose of free drug produced a small and insignificant reduction in survival time. In contrast, whereas the $LD_{50}$ of free actinomycin was 0·43 mg/kg (i.v.) or 0·59 mg/kg (i.p.) no deaths occurred when the liposome-encapsulated drug was given in dose of 1 mg/kg (i.v.) or 2 mg/kg (i.p.). There was thus evidence that liposome incorporation was therapeutically advantageous at the single dose-level tested. Unfortunately, the dose chosen was materially greater than the $LD_{50}$ for the free drug, and smaller doses of the free drug might well have given longer survival, so that this does not

TABLE.—*Toxic and therapeutic effects of WB* 4325 *in oil or in liposomes* (% *in parenthesis*)

| | Dose (mg/kg) | No. treated | Toxic deaths | Progressive growth | Partial response | Complete regression |
|---|---|---|---|---|---|---|
| Oil | 1 | 5 | 0 | 5 | 0 | 0 |
| | 2 | 17 | 0 | 3 | 1 | 13 (76) |
| | 3 | 19 | 0 | 0 | 1 | 18 (95) |
| | 4 | 28 | 1 (4) | 0 | 7 | 20 (71) |
| | 5 | 20 | 8 (40) | 0 | 0 | 12 (60) |
| | 7 | 20 | 9 (45) | 0 | 0 | 11 (55) |
| | 8 | 12 | 11 (92) | 0 | 0 | 1 (8) |
| Liposomes | 10 | 5 | 0 | 1 | 1 | 3 (60) |
| | 18 | 12 | 0 | 1 | 0 | 11 (92) |
| | 30 | 6 | 0 | 0 | 3 | 3 (50) |
| | 41 | 6 | 0 | 0 | 1 | 5 (83) |

constitute a fair comparison of free and entrapped drug.

The study of Kaye *et al.* (1980) did not suffer from this defect. The effects of free and encapsulated actinomycin were measured over a range of doses, and it was found that encapsulation reduced not only lethality but also therapeutic effect (reduction of tumour weight).

Kaye *et al.* (1980) also studied the effects of liposome entrapment on the effect of methotrexate in a range of doses. Both anti-tumour effect and host lethality were increased to the same extent, giving no therapeutic advantage.

An alternative to separate measurements of toxic and therapeutic effects is measurement of the effect of treatment on life-span in animals with lethal tumours. As drug dose increases, survival time first increases due to the anti-tumour effect, reaches a peak, and then falls due to drug toxicity. Thus, therapeutic effect and toxicity are comprehended in the same measurement. This procedure is convenient and is widely used for measuring the mis-called "therapeutic synergy" (Berenbaum, 1981) but it carries a penalty. It may be difficult, and is sometimes impossible, to distinguish with confidence between deaths due to the tumour and those due to toxicity, except near the ends of the dose–response curve. Thus it is not possible to weigh therapeutic effect against toxicity unequivocally, as can be done when they are measured separately. Instead, one treatment is judged to be better than another if its peak survival time or, indeed, any part of its survival curve, is higher than the peak of the other. Evidently, such comparisons cannot be made for a single dose-level of drug (Gregoriadis & Neerunjun, 1975; Mayhew *et al.*, 1976; Kedar *et al.*, 1981).

Measurements of survival time over a range of doses were made by Kobayashi *et al.* (1975), Kimelberg & Atchison (1978) and Ganapathi *et al.* (1980). Kobayashi *et al.* and Ganapathi *et al.* found more or less well-defined peaks in survival time when mice with L1210 leukaemia were treated with cytosine arabinoside in liposomes. However, the doses of free drug were restricted to the rising part of the dose–response curve, and the peak effect was not determined. Thus the conclusion cannot be drawn from these experiments that it is advantageous to give cytosine arabinoside in liposomes.

Kimelberg & Atchison (1978), using methotrexate, studied a sufficiently wide range of doses of free drug and drug in liposomes, but found no therapeutic advantage from liposome incorporation. In fact, i.p. administration of drug-containing liposomes was positively disadvantageous because of increased toxicity.

To sum up, there has hitherto been no good evidence that liposome entrapment of drugs is therapeutically advantageous in treating tumours. In our experiments, we measured toxicity (weight loss) separately from therapeutic effect (tumour regression). Both encapsulated and free drug gave similar maximal rates of complete regression (92% and 95% respectively), but the encapsulated drug did this with negligible toxicity, whereas the free drug did so only at the cost of considerable weight loss. Thus liposome entrapment conferred a clear therapeutic advantage in this case.

Another difference between the two dose–response curves suggests that liposome entrapment may be advantageous, though it does not bear strictly on the question of therapeutic efficiency as defined here. At the optimum dose level of the free drug, a small increase in dose causes deaths from toxicity. In contrast, the optimum dose of the encapsulated drug covers a wide range, which may be even wider than those experiments showed, as increasing the dose caused no material toxicity up to our maximum dose. Thus, in a sense, treatment with the drug in liposomes is a less precarious affair, a consideration that would be clinically important.

Studies of the pharmacokinetics of the drug given in the 2 forms may throw light on the mechanisms of these effects. They are possibly associated with the highly hydrophobic nature of the drug, and investigation of other drugs with similar solubility properties may be rewarding.

We are indebted to the Medical Research Council and Cancer Research Campaign for support, and to Ward Blenkinsop Ltd for a gift of WB 4325.

### REFERENCES

BATZRI, S. & KORN, E. D. (1973) Single layer liposomes prepared with sonication. *Biochem. Biophys. Acta*, **298**, 1015.

BERENBAUM, M. C. (1981) Criteria for analysing interactions between biologically active agents. *Adv. Cancer Res.*, **35**, 269.

BERENBAUM, M. C., COPE, W. A. & DOUBLE, J. A. (1973) The effect of microsomal enzyme inhibition on the immunosuppressive and toxic effects of cyclophosphamide. *Clin. Exp. Immunol.*, **14**, 257.

GANAPATHI, R., KRISHNAN, A., WODINSKY, I., ZUBROD, C. G. & LESKO, L. J. (1980) Effect of cholesterol content on anti-tumor activity and toxicity of liposome-encapsulated 1-β-D-arabinofuranosylcytosine *in vivo*. *Cancer Res.*, **40**, 630.

GREGORIADIS, G. & NEERUNJUN, E. D. (1975) Treatment of tumour bearing mice with liposome-entrapped actinomycin D prolongs their survival. *Res. Commun. Chem. Pathol. Pharmacol.*, **10**, 351.

HOPWOOD, W. J. & STOCK, J. A. (1971) The effect of macromolecules upon the rates of hydrolysis of aromatic nitrogen mustard derivatives. *Chem. Biol. Interact.*, **4**, 31.

KAYE, S. B., BODEN, J. A. & RYMAN, B. E. (1980) The effect of liposome (phospholipid vesicle) entrapment of actinomycin D and methotrexate on the *in vivo* treatment of sensitive and resistant solid murine tumors. *Eur. J. Cancer*, **17**, 279.

KEDAR, A., MAYHEW, E. G., MOORE, R. H. & MURPHY, G. P. (1981) Failure of actinomycin D entrapped in liposomes to prolong survival in renal cell adenocarcinoma-bearing mice. *Oncology.*, **38**, 311.

KIMELBERG, H. K. & ATCHISON, M. L. (1978) Effects of entrapment in liposomes on the distribution, degradation and effectiveness of methotrexate *in vivo*. *Ann. N.Y. Acad. Sci.*, **308**, 395.

KOBAYASHI, T., TSUKAGOSHI, S. & SAKURAI, Y. (1975) Enhancement of the cancer chemotherapeutic effect of cytosine arabinoside entrapped in liposomes in mouse leukemia L1210. *Gann*, **66**, 719.

MAYHEW, E., PAPAHADJOPOULOS, D., RUSTUM, Y. M. & DAVE, C. (1976) Inhibition of tumor cell growth *in vitro* and *in vivo* by 1-β-D-arabinofuranosylcytosine entrapped within phospholipid vesicles. *Cancer Res.*, **36**, 4406.

RAHMAN, Y. E., CERNY, E. A., TOLLAKSEN, S. L., WRIGHT, B. J., NANCE, S. L. & THOMSON, J. F. (1974) Liposome-encapsulated actinomycin D: Potential in cancer chemotherapy. *Proc. Soc. Exp. Biol. Med.*, **146**, 1173.